# Post-Graduation Study

**Published:** 1904-09-03

**Authors:** 


### Post-Graduation Study.

To argue in favour of the provision of special clinical and laboratory facilities for post-graduation study in the several departments of medical work is no longer necessary. The suggestion that the ordinary medical schools provide all that is required, and that special organisations must needs be inferior both in distinction and efficiency, never had any vitality outside a few circles fearful of all new proposals, and to-day it is rarely even heard. Circumstances have made manifest the position, even to those who had not sufficient acuteness to appreciate it in advance, that there is an increasing number of practitioners anxious to obtain opportunities for the enlargement of their professional knowledge and skill, and that such opportunities mean something more than the routine and elementary services planned and conducted for the benefit of the medical student. The demand which is here referred to comes from two different sources. In the first place, the newly-fledged graduate recognises to a much greater extent than was formerly the case that he will do well before settling in practice to equip himself with the more advanced and technical methods of clinical investigation. Possibly, also, he sometimes sees that it may be to his own advantage to acquaint himself with men and manners outside the atmosphere of the institution which has led him through the first stage of his career, and on which he naturally looks with a generous and loyal regard. Certain it is that the compulsory curriculum is often voluntarily extended with benefit both to the young practitioner and his future patients. But it has also to be recognised that there is generally throughout the profession an extended ambition for efficiency and a desire to find opportunities by which this can be gratified. Hence the ranks of the post graduation students are not confined to those fresh from the discipline and triumphs of the schools. They include, in addition, many who have had years of experience in general practice and who are anxious to bring their knowledge and work up to the highest attainable level. There can be no question that in all this there are great possibilities for good, both for the science and art of medicine and for the public interest. It offers indeed one of the most encouraging prospects for all those who are concerned for the welfare of the medical profession and for the service it is capable of rendering to mankind. It may be, indeed it certainly is, the case, that there has been a lamentable want of foresight on the part of many in positions of authority and influence in failing to appreciate the necessity for encouraging and meeting the demand for post-graduation study. But the movement has now reached a secure position and is safe even from the superior person.

It is inevitable that in the development of this movement London must sooner or later occupy a leading position. Apart from all other considerations there is no other city which can offer so rich and varied a clinical field, and hence it is satisfactory to note that of recent years the cause of post-graduation instruction in the metropolis has received wide recognition, and its principles and necessary conditions have been included in definite organisations devoted to this particular purpose. As already suggested one of these conditions is that the opportunities offered to the practitioner or graduate must be something different from those supplied in the education of the medical student. No one at all familiar with the question will dispute this proposition, and hence it is satisfactory to find that the two principal institutions in London which profess to supply these opportunities confine themselves entirely to post-graduation instruction and do not admit to their classes those who have a medical diploma or qualification yet to gain. These institutions are the West London Hospital and College and the Medical Graduates' College and Polyclinic.

### The West London Hospital and College.

The West London Hospital is a fully equipped general hospital having 159 beds and an out-patient department at which some 30,000 new patients are seen every year. In addition to the general physicians and general surgeons the staff includes representatives of all branches of special practice, each of whom conducts an appropriate clinical service at the hospital. The whole of this practice, both indoor and outdoor, is utilised for purposes of clinical teaching. The college in connection with the hospital has been gradually extended and developed so that it may now claim to be a thoroughly well organised and well-equipped institution; recently there has been added a new pathological laboratory, and the arrangement for teaching microscopy and bacteriology have been brought well up-to-date. Lectures on medicine, surgery, etc., are given daily by members of the staff, and, for those who desire it, special instruction is provided with a view to prepare candidates for the higher examinations. There is ample provision for the convenience of practitioners attending the College in the shape of reading-room, smoking-room, library, etc., and though Hammersmith, where the hospital is situated, is not exactly in the centre of London, the facilities for travel will satisfy the most exacting. The fees for hospital practice, including all the ordinary demonstrations and lectures, are £2 2s. for one month, £4 4s. for three months, £6 6s. for six months, and £9 9s. for 12 months. To put the position in brief, it is certain that for these fees the practitioner has every chance of following under experienced teachers the practice of a fairly large modern hospital, and, in view of these advantages, the charges are obviously moderate.

### The Medical Graduates' College and Polyclinic.

The Medical Graduates' College and Polyclinic (22 Chenies Street, Gower Street, W.C.) has a special position, inasmuch as it does not draw its teachers from the staff of any one hospital, but its work is conducted by lecturers and demonstrators from many schools. Hence, within its walls may be heard the teaching of many men of the highest possible repute. The special clinical service exists in the form of a daily demonstration (at 4 p.m.) at which selected cases are exhibited and discussed. On most days of the week, too, there is a clinical lecture or lantern demonstration. These are open to all registered practitioners for the annual fee of £1 1s., which also includes the use of the museum, library, reading-room, etc, and the receipt of the *Polyclinic*

*Journal.* This latter is issued every month, and contains an account of many of the cases exhibited at the clinical demonstrations. The museum contains a rich collection of drawings, photographs, models, etc., presented by Mr. Jonathan Hutchinson, who conducts a clinique at the college every Thursday afternoon. There is also a thoroughly efficient clinical and bacteriological laboratory, and special practical classes are arranged in ophthalmology, laryngology, otology, *x* ray work, neurology, etc. For each of these the fee is £1 1s There are already about 1,000 members of the Polyclinic, and the value of the institution as a field for clinical study is being recognised to an increasing extent.

Charing Cross, Westminster, and Tottenham Hospitals.

In addition to the above, special clinical courses are arranged for practitioners at the Charing Cross, Westminster, and Tottenham Hospitals.

London Post Graduate Association.

Another scheme is that arranged by the so-called "London Post-Graduate Association," which includes 10 of the leading general metropolitan hospitals, together with several special hospitals. A fee of five guineas admits the holder to the ordinary practice and teaching of these for a period of three months. Applications should be addressed to the Secretary, Examination Hall, Victoria Embankment, W.C.

It has also to be noted, and not without gratification, that several of the provincial towns having medical schools have recently organised post-graduation teaching in various departments, and the advantages to practitioners in their neighbourhoods are obvious. But for those who can give the whole of their time for a month or longer, London can offer special opportunities, and practical hints for guidance can readily be obtained from the officials of the leading post-graduation colleges.

